# Inpatient Small Bowel Capsule Endoscopy: Not Associated With Bleeding Site Identification or 30-Day Readmission Prevention

**DOI:** 10.7759/cureus.74043

**Published:** 2024-11-19

**Authors:** Ismail Ghafary, Talal Seoud, Michael Jorgensen, Jade Marhaba, William M Briggs, Daniel S Jamorabo

**Affiliations:** 1 Gastroenterology and Hepatology, University of Connecticut School of Medicine, Farmington, USA; 2 Gastroenterology and Hepatology, University of Florida, Gainesville, USA; 3 Internal Medicine, Stony Brook University Hospital, Stony Brook, USA; 4 Gastroenterology and Hepatology, Stony Brook University Hospital, Stony Brook, USA; 5 Biostatistics, NewYork-Presbyterian, New York, USA; 6 Gastroenterology and Hepatology, Stony Brook Medicine, Stony Brook, USA

**Keywords:** gastrointestinal bleeding, gi endoscopy, hospital readmission, inpatient care, small bowel capsule endoscopy

## Abstract

Background

The utility of small bowel capsule endoscopy (SBCE) in the inpatient setting is controversial due to retention rates and costs.

Aim

This study aims to evaluate whether using SBCE significantly improved the identification of potential bleeding sites or reduced the risk of 30-day readmission for overt or occult gastrointestinal bleeding.

Methods

This was a single-center retrospective cohort study involving inpatients who underwent SBCE at a suburban tertiary care hospital from January 1, 2012, to January 1, 2022, for suspected small bowel bleeding. There was no control group used in this observational study. We used chi-square testing to determine the significance among our categorical variables and t-tests to compare means for our numerical variables. We also did multivariable logistic regression to analyze risk factors for increased hospital stay. All statistical analysis was done in R (R Core Team, 2020, R Foundation for Statistical Computing, Vienna, Austria).

Results

We identified 514 inpatients who underwent SBCE from January 1, 2012, to January 1, 2022, including 300 (58.4%) men and 214 (41.6%) women. Most (305/514, 59.3%) had no notable findings on SBCE, but 209/514 (40.7%) subsequently underwent endoscopic procedures, and a bleeding site was identified and treated in 168/209 (80.4%). Undergoing a subsequent procedure significantly increased the average number of days between capsule deployment and discharge (9.6 vs. 4.9 days, p < 0.005) without significantly reducing the risk for 30-day readmission (OR 1.33, 95% CI 0.9-1.9, p = 0.2). Among the 209 patients who had a subsequent procedure, identifying and treating a bleeding site did not significantly change readmission rates (OR 1.35, 95% CI 0.6-3.1, p = 0.5) compared to patients who did not have a procedure.

Conclusion

We did not find that inpatient SBCE significantly affected 30-day readmission rates even if an endoscopic procedure was subsequently done or a potential bleeding site was treated.

## Introduction

Small-bowel bleeding, defined as bleeding originating between the ampulla of Vater and the ileocecal valve, poses a diagnostic challenge since it is not easily located with conventional upper and lower endoscopic evaluations [1]. This subset of patients constitutes approximately 5% of hospital admissions for gastrointestinal (GI) bleeding and imposes a financial strain on the healthcare system with an annual cost burden of $10,000-$15,000 per patient and readmission rates exceeding 25% [2]. Current guidelines from major gastroenterology societies recommend small bowel capsule endoscopy (SBCE), also known as video capsule endoscopy (VCE), as the subsequent diagnostic modality for this patient cohort [3]. Although outpatient SBCE utility has been extensively studied, the literature relating to the efficacy of inpatient SBCE remains limited.

Inpatient SBCE usage can involve several obstacles, including suboptimal bowel preparation and incomplete small bowel examination, thereby resulting in diminished diagnostic efficacy [3-7]. Obesity has also been associated with reduced small bowel transit time and lower SBCE yield [8]. Concerns regarding capsule retention persist, with reported incidence rates ranging from 1.4% to 5.3% [9-12]. Despite advancements in technology, studies indicate comparable rates of recurrent bleeding regardless of SBCE findings and subsequent push enteroscopy [13-15]. Moreover, the timing of SBCE procedures can significantly influence diagnostic yield, with examinations conducted more than three days after presentation showing a notable decline in efficacy [16,17]. Additionally, evidence suggests that a subset of patients undergoing SBCE harbors lesions within reach of conventional endoscopic techniques [18].

In this study, we sought to determine whether inpatient SBCE deployment facilitated the identification and treatment of small intestine bleeding sources and prevented 30-day hospital readmission.

## Materials and methods

This is a single-center retrospective cohort study involving inpatients who underwent SBCE from January 1, 2012, to January 1, 2022. 

Study design and population

We identified adults aged 18 years and over who were admitted to our tertiary-care academic center and who underwent SBCE during their hospitalization from January 1, 2012, to January 1, 2022. All patients provided written informed consent for SBCE. Inclusion criteria included patients with upper endoscopy and colonoscopy unrevealing for a source of anemia. No control group was used since this was a retrospective cohort study. Exclusion criteria included patients with a history of small bowel surgery or known small bowel pathology. A total of 514 patients underwent SBCE to investigate obscure gastrointestinal bleeding (OGIB) at our institution when filtered by the criteria cited. The study was approved by the Stony Brook University Institutional Review Board (IRB# 2021-00530).

Data collection

A chart review was conducted to collect demographic information on all patients, including sex, age, documented past medical history, initial hemoglobin level, incidence of rebleeding (defined as a decrease in hemoglobin level less than 2 g/dL, recurrent melena or hematochezia), anti-platelet use, and anticoagulant use. All information was de-identified. 

VCE protocol and review

Our institution used the Medtronic PillCam (Model SB/SB2; Given Imaging Ltd., Yokne'am Illit, Israel) throughout the study period. Capsules were deployed orally or endoscopically at the attending gastroenterologist's discretion. Patients could start taking clear liquids by mouth two hours after capsule deployment. All patients had nothing by mouth for 12 hours prior to capsule deployment and had a bowel preparation consisting of 238 g of polyethylene glycol mixed in 2 L of water. Patients whose SBCE examination was incomplete, with the capsule not reaching the cecum within the battery time, were excluded. All videos were reviewed with conventional lighting by an experienced central investigator with over 300 previous capsule studies performed. The images were reviewed at a maximum of 12 frames per second. All findings were labeled either as P1 lesions (uncertain potential bleeding such as erosions or red spots) or as P2 lesions with a high potential for bleeding such as ulcers, angioectasias, varices, or tumors).

Statistical analysis

We used chi-square testing to determine the significance among our categorical variables and t-tests to compare means for our numerical variables. We also did multivariable logistic regression to analyze risk factors for increased hospital stay. All statistical analysis was done in R (R Core Team, 2020, R Foundation for Statistical Computing, Vienna, Austria). A p-value of less than 0.05 was considered statistically significant. The baseline quantitative data are presented as mean ± SD.

## Results

Baseline characteristics

A total of 514 inpatients who underwent SBCE between January 1, 2012, and January 1, 2022, were included in the analysis. The mean age of the cohort was 73 years, with a predominance of male patients (300/514; 56.4%), and the majority identified as white non-Hispanic individuals (456/514; 88.7%) (Table 1). The socioeconomic status of most patients fell within the categories of Comfortable or Prosperous based on The Distressed Communities Index (DCI) (80.9%), which is used by the federal government to measure economic well-being at the zip code level based on local unemployment and poverty rates, median income, and other socioeconomic indicators. Documented coronary artery disease was the most prevalent comorbidity among the cohort, affecting 59.7% (307/514) of patients. Anemia constituted the primary indication for SBCE in most cases (480/514; 93.4%), followed by melena (225/514; 43.8%) and hematochezia (100/514; 19.5%). A minority of patients presented with symptoms suggestive of occult GI bleed (201/514; 39.1%). Notably, a significant proportion of patients (340; 66.1%) achieved resolution of overt bleeding prior to capsule deployment.

**Table 1:** Baseline characteristics for cohort

Variable	Value
Total, N	514
Mean age (standard deviation)	73.3 (12.7)
Mean body mass index (standard deviation)	29.3 (6.54)
Number of women (%)	214 (41.6)
Number of men (%)	300 (58.4)
Race, White, N (%)	456 (88.7)
Comorbidities	N (%)
Cardiomyopathy (CM)	192 (37.4)
Coronary artery disease (CAD)	307 (59.7)
Chronic kidney disease (CKD)	134 (26.1)
Chronic obstructive pulmonary disease (COPD)	115 (22.4)
Diabetes mellitus (DM)	153 (29.8)
None	99 (19.3)
Indication for video capsule endoscopy:	N (%)
Melena	225 (43.8)
Anemia	480 (93.4)
Hematochezia	100 (19.5)
Abdominal pain	25 (4.8)
Resolution of overt bleeding prior to capsule deployment, N (%)	340 (66.1)
On antiplatelet or anticoagulation prior to hospital admission, N (%)	351 (68.4)
Aspirin	236 (45.9)
Clopidogrel	91 (17.7)
Prasugrel	3 (0.58)
Brilinta	11 (2.14)
Apixaban	60 (11.7)
Rivaroxaban	27 (5.25)
Dabigatran	4 (0.778)
Edoxaban	2 (0.389)
Warfarin	108 (21)
Unfractionated heparin	1 (0.195)
Enoxaparin	7 (1.36)

The use of antiplatelet (AP) or anticoagulant medications was common among the cohort, with 68.4% (351/514) of patients receiving such therapy prior to hospital admission. Aspirin was the most used anticoagulant (236/514; 45.9%).

Findings and subsequent procedures

A total of 514 patients underwent SBCE. The SBCE findings were categorized using the Saurin classification (Table 2). The distribution of findings was as follows: 322 patients (62.6%) had no findings, 24 patients (4.7%) had findings with no potential of bleeding (P0), no patients (0%) had findings with low/uncertain potential of bleeding (P1), and 168 patients (32.7%) had findings with a high potential of bleeding (P2). The P2 class contained 35 patients with ulcers, four patients with masses, and 132 patients with arteriovenous malformations (AVMs)/angioectasias. Out of the 514 patients, 133 (25.9%) were readmitted for bleeding. Among these readmissions, the distribution by SBCE finding type was as follows: 76 patients (14.8%) had no findings, six patients (1.2%) had findings with no potential of bleeding (P0), no patients (0%) had findings with low/uncertain potential of bleeding (P1), and 51 patients (9.9%) had findings with a high potential of bleeding (P2).

**Figure 1:**
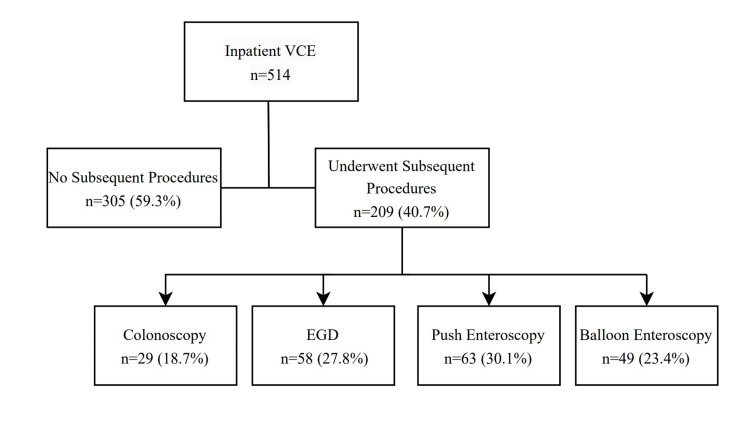
Procedures following video capsule endoscopy (VCE) EGD, esophagogastroduodenoscopy

Among the 514 patients who underwent SBCE, subsequent endoscopic procedures were indicated in 209 patients (40.7%) (Figure 1), with a bleeding site identified and treated in 168 of these cases (80.4%). Of the 209 patients who underwent subsequent procedures, 45.9% experienced readmission due to bleeding. The odds ratio (OR) for readmission within 30 days following subsequent procedures was 1.33 (95% confidence interval (CI) 0.9-1.9, p = 0.2), suggesting no significant change in readmission risk when patients had a procedure after SBCE deployment.

Even among patients in whom a bleeding site was found and treated, there was no significant change in 30-day readmission rates (OR 1.35, 95% CI 0.6-3.1, p = 0.5) or in readmission time after discharge (27 vs. 35 days, p = 0.09) compared to individuals in whom a bleeding source was not found. Additionally, there was no difference in readmission rates when comparing patients with no lesions found on SBCE (76/322) or lesions of no bleeding potential (P1) (6/24) to patients with lesions of high bleeding potential (P2) (51/168) (Table 2).

**Table 2:** Small bowel capsule endoscopy (SBCE) findings and readmission by SBCE findings

Findings	
Total, N	514
Video capsule endoscopy findings (Saurin classification)	N (%)
No gastrointestinal bleeding site visualized	322 (62.6)
P0 (findings with no potential of bleeding)	24 (4.7)
P1 (findings with low/uncertain potential of bleeding)	0 (0)
P2 (findings with high potential of bleeding)	168 (32.7)
Readmission for bleeding by video capsule endoscopy finding type	133 (25.9)
No gastrointestinal bleeding site visualized	76 (14.8)
P0 (findings with no potential of bleeding)	6 (1.2)
P1 (findings with low/uncertain potential of bleeding)	0 (0)
P2 (findings with high potential of bleeding)	51 (9.9)

Patients undergoing subsequent procedures experienced a significant increase in the average number of days between capsule deployment and discharge (9.6 vs. 4.9 days, p < 0.005) and the number of hours between capsule read and discharge (215 hours vs. 115 hours, p < 0.005). Multivariable analysis revealed a higher probability of requiring subsequent procedures for patients with anemia (2.3-fold, p = 0.04) and melena (2.1-fold, p < 0.0001), while resolution of overt bleeding and stable hemoglobin levels pre-SBCE were associated with a lower such probability (0.28-fold, p < 0.0001 and 0.98-fold, p < 0.0001, respectively).

Patients on anticoagulation (AC) or AP therapy prior to hospital admission were significantly older than patients who were not on either medication (75.2 years old vs. 69.1 years old, p < 0.0001). Otherwise, being on AC or AP was not associated with DCI status, body mass index, stable hemoglobin, prolonged stay, or increased risk for readmission. On multivariable analysis, patients on AC or AP prior to hospital admission had a significantly increased risk for anemia and melena (OR 3.66, 95% CI 1.8-7.7, p < 0.001; OR 1.63, 95% CI 1.1-2.4, p < 0.05, respectively), though with a significantly decreased risk for hematochezia (OR 0.45, 95% CI 0.3-0.7, p < 0.001).

Discharge and readmission

**Figure 2:**
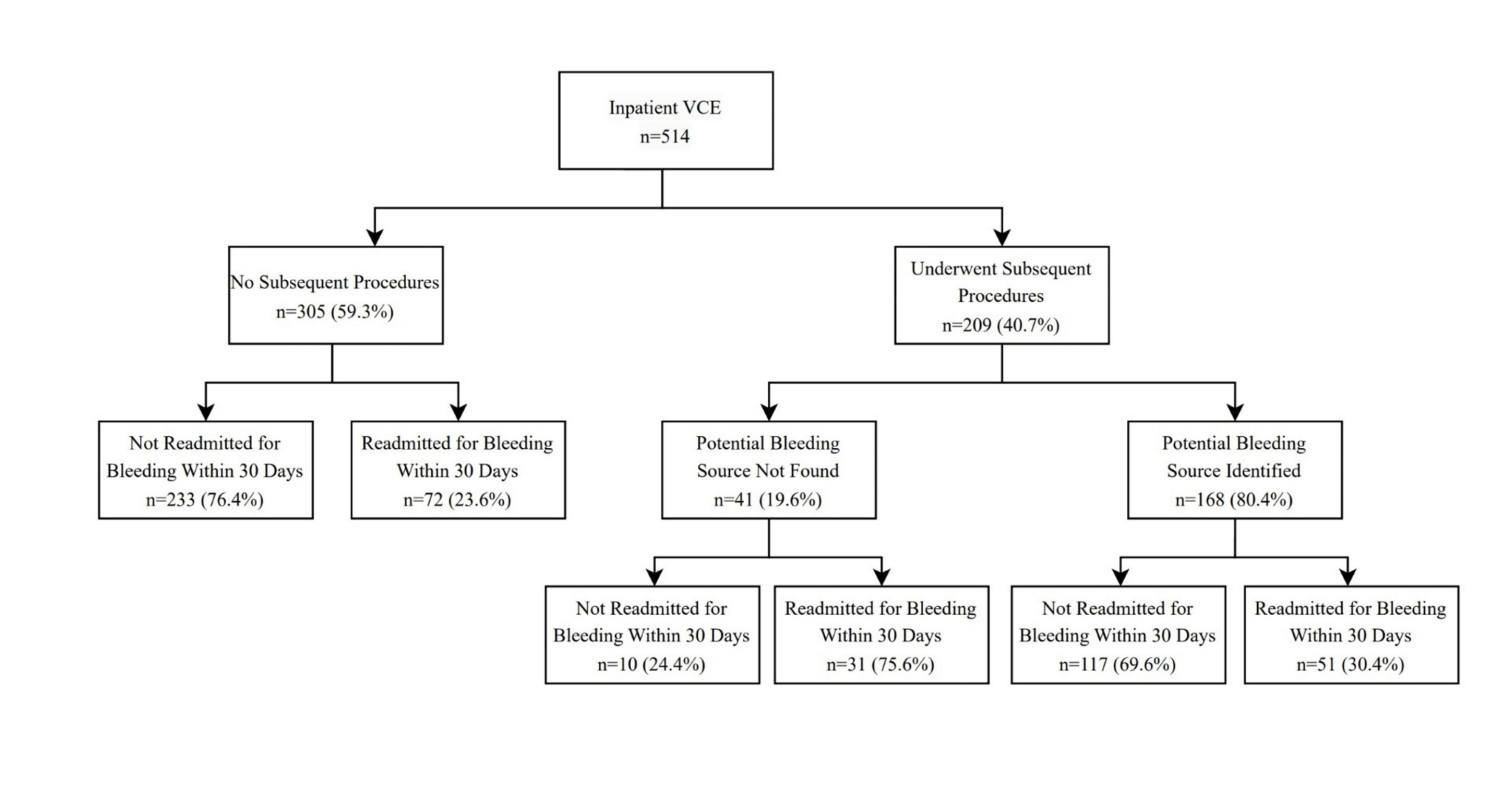
Readmission rates 30 days after inpatient intervention

There was no significant difference between White and non-White patients in hemoglobin stability pre-SBCE, number of days between SBCE deployment and discharge, time to discharge after SBCE read, or readmission time after discharge (Figure 2).

Multivariable analysis revealed no variable to be significantly associated with 30-day readmission. The mean duration of stable hemoglobin prior to capsule deployment was 39.6 hours (SD 22.7), with an average duration between capsule deployment and discharge of 6.89 days (SD 12.4). The mean time of discharge after capsule read was 6.5 days (SD 12 days), and the mean readmission time after discharge was 31.7 days (SD 30). A total of 133 patients (SD 25.9) were readmitted for recurrent bleeding. Patients receiving AC or AP therapy prior to admission did not exhibit a significantly increased risk of readmission after discharge compared to those not on such therapy (OR 1.32, 95% CI 0.9-2.0, p = 0.22).

## Discussion

Current guidelines from major gastroenterology societies recommend the use of SBCE to evaluate for a potential small bowel source following a negative upper and lower endoscopy in patients with GI bleeding [1,19,20]. Although this may help provide further diagnostic capabilities, the healthcare cost and lack of clear benefits are a significant concern [21]. Our study further supports this finding. Strengths of this study include the large sample size of 514 patients and the extended observation period of 10 years. We found that in hospitalized patients with suspected GI bleeding who underwent SBCE, a subsequent procedure significantly increased hospital length of stay, without significantly reducing readmission risk or time after discharge. This remained true even if a bleeding site was identified and treated. The use of AC did not increase the risk of readmission for GI bleeding either. Our findings question the utility and cost-effectiveness of inpatient SBCE use since we did not find that 30-day readmission rates for suspected bleeding were affected, even if an endoscopic procedure was subsequently done or a potential bleeding site was treated. Despite the disagreement between the current literature and our results, we believe it is important to share these limitations of SBCE with the gastroenterology community.

As SBCE lacks therapeutic options, deep enteroscopy with either single-balloon enteroscopy (SBE) or double-balloon enteroscopy (DBE) is often required after a positive diagnostic result on the VCE exam. Deep enteroscopy is time-consuming and requires facility resources with extensive training and time allotment, which may not be feasible in a busy academic setting [22,23]. Patients with altered anatomy may be at increased risk for perforation or incomplete examination [17,24]. Therapeutic endoscopic yield has been shown to be higher if performed sooner after a bleeding episode, but that can be costly and logistically impractical [25-27]. SBE and DBE therapeutic yield have been proposed to be increased by examination under anesthesia and within 24 hours of presentation, a resource-heavy and challenging metric to meet in many situations [28]. 

SBCE and DBE results have also been found to be suboptimal in agreement [29-31]. SBE and DBE enteroscopy have diagnostic and therapeutic rates that range from 60% to 80% [32]. Early enteroscopy after positive SBCE has not been found to correlate with increased endoscopic yield, likely due to the typical time delay between procedures [33,34]. DBE has not been found to alter the risk of recurrent bleeding in many patient populations [35-40]. Mortality rates have been found to be the same upon follow-up regardless of whether the patient presents with isolated or non-isolated small bowel angiodysplasias [41]. DBE has also been found to have a lower yield for OGIB compared to other indications for the procedure [42]. It has been shown that a suspected source of bleeding could be identified on colonoscopy and examination of the TI [43], potentially removing the need for SBCE. Analysis of new models of SBCE, including different generation and brand iterations, have shown nonsignificant differences in diagnostic yield, suggesting a possible plateau to this technology’s potential detection capability [44]. Finally, reading inpatient capsule studies can take upward of an hour per study and requires familiarity, training, and widespread adoption [45,46]. 

It is important to acknowledge that there is a discrepancy between our study findings and a recent meta-analysis that examined re-bleeding rates after SBCE for OGIB among Western and Eastern populations [47]. The meta-analysis concluded that the odds of re-bleeding were significantly higher after positive index SBCE in Eastern populations and that application of specific treatment after positive index SBCE was associated with lower re-bleeding odds. We do not believe these findings are entirely applicable to our study given the geographic and ethnic differences in our patient population in addition to our retrospective designs being limited to 10 years at one academic institution in the United States. 

We recognize that there are scenarios in which inpatient SBCE use has shown utility. In patients with obscure GI bleeding, for example, one study showed a high rate of correlation between capsule endoscopy and subsequent procedures, thus assisting in diagnosis [48]. Additionally, the timing of capsule deployment is tied strongly to its ability to identify a bleed. Another team showed that patients who underwent SBCE less than 48 hours after their last OGIB had higher diagnostic utility, intervention rates, and shorter hospital stays compared to those who underwent SBCE after 48 hours [49]. Early capsule deployment and increased bleeding localization were also seen in one randomized control trial, which compared an early capsule arm (capsule deployment within ten minutes of consent) to a standard of care arm (timing based on the consultant gastroenterologist’s discretion) [50]. However, in practical scenarios, capsules may not be typically deployed early enough in a patient’s hospital course to change overall management.

The integration of artificial intelligence (AI) holds promise in addressing certain limitations associated with SBCE, potentially reducing physician reading times and augmenting the diagnostic yield of SBCE findings [46]. Ongoing investigations continue to evaluate the efficacy of the latest iterations of SBCE technology in detecting small bowel bleeding. Moreover, research endeavors are expanding to explore the broader applications of SBCE technology, including its potential as a viable alternative to conventional colonoscopy for routine cancer screening. Magnet-controlled capsule technology represents a notable advancement in SBCE technology, offering the prospect of more comprehensive examinations and broader applicability [21]. 

Limitations

This study is a retrospective analysis, which is inherently susceptible to selection bias. The study is also confined to a single large tertiary medical center in the United States, which may limit the generalizability of our findings to broader patient populations seen in multi-center settings. Moreover, our tertiary medical center is geographically situated amongst other community-based hospitals, where patients might have sought care for subsequent bleeding episodes. As a result, this could have led to an underestimation of the true readmission rate captured in our study. Direct comparisons may not be accurate in this study due to the absence of randomization and variability of lesions identified with differing chances of rebleeding. Additionally, the lack of statistical difference does not necessarily mean a change in a patient’s prognosis. Given the 10-year timeline of our study data, there is a high risk of variability in the interpretation of the capsule studies by different gastroenterologists, recording times, and image quality of the studies. Furthermore, our investigation is a retrospective study, which is inherently susceptible to selection bias.

SBCE technology has inherent limitations that may prevent its widespread adoption. Reader fatigue has been shown to decrease the diagnostic yield of VCE, along with a general lack of reader experience or confidence [9]. Complete visualization of the small bowel is not always achieved, with inpatient status a potential confounding factor [10]. There is also the added expense that hospitals acquire by performing these procedures, particularly in an inpatient setting where reimbursement may be lower when compared to an outpatient setting [22].

## Conclusions

In this large, decade-long study of 514 patients, we found that inpatient SBCE did not significantly reduce 30-day readmission rates, even when bleeding sources were identified and treated. Subsequent procedures following SBCE increased the length of stay without improving outcomes, raising questions about the cost-effectiveness of inpatient SBCE deployment. These findings suggest that current guidelines regarding inpatient SBCE use may need reassessment, particularly considering healthcare resource utilization. We still believe that SBCE can be useful in the inpatient setting and should be utilized on a case-by-case basis after considering hemodynamic status, patient compliance, goals of care, the volume of blood lost, and overall clinical prognosis, among other factors. Prospective, randomized trials comparing outcomes with early outpatient versus inpatient SBCE deployment, or else prospective inpatient cohort studies wherein patients get early SBCE deployment, are needed to establish optimal protocols that balance patient outcomes with healthcare resource utilization.
